# Evaluation of resistance and determination of stability of different sugar beet (*Beta vulgaris* L.) genotypes in rhizomania‐infected conditions

**DOI:** 10.1002/fsn3.3180

**Published:** 2022-12-20

**Authors:** Abazar Rajabi, Masoud Ahmadi, Mohsen Bazrafshan, Mehdi Hassani, Ali Saremirad

**Affiliations:** ^1^ Sugar Beet Seed Institute (SBSI) Agricultural Research, Education and Extension Organization (AREEO) Karaj Iran; ^2^ Khorasan Razvi Agricultural and Natural Resources Research and Education Center Agricultural Research, Education and Extension Organization (AREEO) Mashhad Iran; ^3^ Fars Agricultural and Natural Resources Research and Education Center Agricultural Research, Education and Extension Organization (AREEO) Shiraz Iran; ^4^ Hamedan Agricultural and Natural Resources Research and Education Center Agricultural Research, Education and Extension Organization (AREEO) Hamedan Iran

**Keywords:** adaptation, gene, infection, race, susceptibility

## Abstract

Plant diseases are considered one of the main factors reducing yield and quality of crops, which are constantly developing and creating more virulent races and cause the resistance of more genes to break. Identifying resistance sources and including them in breeding programs will improve resistant genotypes. Rhizomania is the most common, widespread, and devastating disease of sugar beet in Iran and worldwide. Breeding genotypes with disease resistance genes is one of the most important ways to deal with this destructive disease. Twenty sugar beet genotypes along with five controls were evaluated in a randomized complete block design with four replications in rhizomania‐infected conditions in four regions of Mashhad, Shiraz, Miandoab, and Hamedan for 2 years. The results of genotypic reaction to rhizomania showed that the genotypes with resistance reaction were much more frequent than those with susceptibility reaction. The analysis of multiplicative effects of the AMMI model showed that the first six components were significant and explained 98.80% of the interaction variations. The biplot obtained from the mean white sugar yield and the first interaction principal component confirmed the superiority of the RM5 genotype due to its high white sugar yield and stability in infected conditions. The results obtained from the first three principal components biplot showed that the RM9 genotype with a mean white sugar yield of 11.91 t. ha^−1^ was a genotype with vast general stability in all disease‐infected environments. Based on the results of the MTSI index, RM3, RM17, RM9, RM13, and RM15 are introduced as stable genotypes under rhizomania‐infected conditions. In conclusion, it seems that the studied genotypes have valuable and useful genes inherited from their parents to deal with rhizomania disease. Applying these genotypes in sugar beet breeding programs can effectively prevent the threat of rhizomania.

## Graphical abstract

Rhizomania is the most common, widespread, and devastating disease of sugar beet in Iran and worldwide. Breeding genotypes with disease resistance genes is one of the most important ways to deal with this destructive disease. Twenty sugar beet genotypes with five controls were evaluated in natural infection conditions to rhizomania diseases in four regions of Mashhad, Shiraz, Miandoab, and Hamedan in a randomized complete block design with four replications for 2 years.

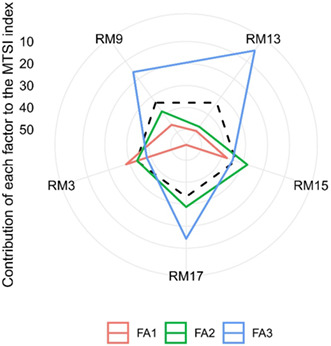

## 1 INTRODUCTION

According to United Nations (2019) investigations, the world population is expected to increase from 7.7 billion people in 2019 to 8.5 in 2030, 9.7 in 2050, and 10.9 in 2100. Naturally, with the growth of population, the demand for food will increase significantly. Sugar is a common molecule that has been of special nutritional importance in all eras (Eggleston, 2019). This molecule acts as a tonic matter and provides a large part of the energy in the human diet. Sugar beet is an important agricultural product which is exclusively used in the sugar industry (Akyüz & Ersus, 2021) and is considered one of the most important sources of sugar production after sugar cane (Monteiro et al., 2018; Ribeiro et al., 2016) so that currently, it accounts for 20%–30% of the world sugar production (Iqbal & Saleem, 2015; Monteiro et al., 2018; Ribeiro et al., 2016). Sugar is the main product of sugar beet, but, in addition, many other by‐products such as molasses, marc, and ethyl alcohol are also extracted from this plant during the sugar production process (Tomaszewska et al., 2018). In addition, the leaf of this plant has protein compounds (Lammens et al., 2012; Tenorio et al., 2017) and balanced amino acids (Akyüz & Ersus, 2021; Kiskini et al., 2016), which reveals the nutritional quality of sugar beet leaves. Therefore, considering the importance of sugar beet in the nutrition of human societies, it is necessary to pay attention to its yield and quality.

Agricultural production is influenced by biotic and abiotic stresses. These factors challenge the plant's quantitative and qualitative production and destroy a considerable part of agricultural products annually. Diseases and pests are among the most important biotic stresses that affect both quantitative and qualitative aspects of plant products (Abdallah et al., 2003; Oerke, 2006). Sugar beet (*Beta vulgaris* L.) is no exception to this rule and is influenced by pathogens. Rhizomania is one of the most common diseases of sugar beet in the world (Galein et al., 2018), which can cause a sharp decrease in yield and product quality (Rezaei, 2007). The disease is caused by *Beet Necrotic Yellowing Vein Virus* (BNYVV) and its vector is *Polymyxa betae* Keskin (Tamada, 1975; Tamada & Baba, 1973), which has potentially been a destructive disease of sugar beet on a global scale (McGrann et al., 2009) in such a way that the amount of damage caused by it may lead to the destruction of sugar beet fields on a large scale. The first report of the disease published in the world was related to northern Italy in 1952 (Pavli et al., 2011). This disease in Iran was reported for the first time in Fars province by Izadpanah et al. (1996); subsequently, its existence was proved in sugar beet fields of most parts of the country (Arjmand & Ahun Manesh, 1996). Of course, the spread of rhizomania is not unique to Iran, and currently, this disease is known as the most important cause of damage in sugar beet fields in most countries of the world (Galein et al., 2018; Lennefors, 2006; Tamada, 1999). Therefore, if effective methods are not used to control it, it will significantly reduce crop yield and quality (Rezaei, 2007).

Genetic, chemical, agronomical, and biological control methods have been presented against rhizomania disease. Among the mentioned methods, agronomical and biological methods are not very important due to their low ability to control the disease and compared to the genetic and chemical control methods, they are less used. Control of the disease by chemical compounds is not always practical due to the high cost, the risk of the developing of pathogen's resistance to it, and the possibility of environmental pollution; On the other hand, due to the soil‐borne nature of rhizomania disease and the ineffectiveness of conventional methods of combating soil‐borne diseases, the pioneers of breeding disease‐resistant varieties consider genetic resistance as the most effective way to reduce the damage caused by such diseases. In this regard, it is most important to investigate the amount of genetic diversity in cultivars and lines and carry out genetic studies to identify and select disease‐resistant genotypes; Therefore, the present study was conducted to evaluate sugar beet genotypes in terms of resistance to rhizomania disease and yield stability to use resistant genotypes in breeding programs and also to introduce them for cultivation in infected environments.

## 2 MATERIALS AND METHODS

### 2.1 Plant materials and details of experiments

A set of 20 genotypes along with domestic resistant control of SBSI038 and four foreign‐resistant controls of Lexia, Baloo, Poseidon, and BTS335 formed the present study's genetic material (Table 1). The plant materials were cultivated in a randomized complete block design with four replications in each of the four agricultural research stations of Mashhad, Shiraz, Miandoab, and Hamedan, Iran. These research stations are naturally infected with rhizomania disease. To ensure the infection of the experiments with rhizomania, the Sharif susceptible variety was cultivated around the experiments. The specifications of the research stations are presented in Table 2.

**TABLE 1:** Specifications of the studied sugar beet genotypes

Genotype code	Origin	Genotype code	Origin	Genotype code	Origin
RM1	F‐21076	RM10	F‐21085	RM19	F‐21095
RM2	F‐21077	RM11	F‐21086	RM20	F‐21096
RM3	F‐21078	RM12	F‐21087	SBSI038	Domestic check
RM4	F‐21079	RM13	F‐21088	Lexia	Foreign check
RM5	F‐21080	RM14	F‐21089	Baloo	Foreign check
RM6	F‐21081	RM15	F‐21090	Poseidon	Foreign check
RM7	F‐21082	RM16	F‐21091	BTS335	Foreign check
RM8	F‐21083	RM17	F‐21092	‐	‐
RM9	F‐21084	RM18	F‐21093	‐	‐

**TABLE 2:** Geographical characteristics of the experimental research stations

Environment code	Location of the research station	Altitude (m)	Latitude	Longitude
E1	Mashhad, Khorasan Razavi, Iran	1316	36°30' N	59°37′ E
E2	Shiraz, Fars, Iran	1484	29°32' N	52°36′ E
E3	Miandoab, West Azerbaijan, Iran	1296	36°58' N	46°05′ E
E4	Hamedan, Iran	1818	34°47' N	48°30′ E

### 2.2 Disease assessment and measurement of quantitative and qualitative root traits

The disease severity was recorded at harvest stage according to the Luterbacher et al. (2005) method on a scale of 1–9 at two agricultural research stations in Mashhad and Shiraz. The scores 1 and 9 represent the lowest and highest disease infection, respectively. Although the experiments were also conducted at Miandoab and Hamadan agricultural research stations under disease‐infected conditions, the data on the infection severity were not recorded in these two stations.

After harvesting and recording of disease severity and root yield, the roots were washed and a pulp sample was randomly prepared from the roots of each plot; the pulp samples were then examined at the quality control laboratory for quality characteristics including sugar content, alpha‐amino N, sodium (Na^+^), and potassium (K^+^) elements (Kunz et al., 2002). Finally, the obtained values were used to estimate other characteristics such as sugar yield, molasses sugar, white sugar content, white sugar yield, and extraction coefficient of sugar, based on Equations (1), (2), (3), (4), (5), respectively (Cook & Scott, 1993; Reinfeld et al., 1974).

(1) SY=RY×SC

(2) $$\mathrm{MS}=0.0343\left(K^{+}+{\mathrm{Na}}^{+}\right)+0.094\left(\text{alpha amino}\;N\right)-0.31$$

(3) $$\mathrm{WSC}=\mathrm{SC}-\left(\mathrm{MS}+0.6\right)$$

(4) WSY=WSC×RY

(5) $$\mathrm{ECS}=\left(\frac{\mathrm{WSC}}{\mathrm{SC}}\right)\times 100$$

where SY is sugar yield (t. ha^−1^), RY is root yield (t. ha^−1^), SC is sugar content (%), MS is molasses sugar (%), K^+^ is potassium (meq.100 g^−1^), Na^+^ is sodium (meq.100 g^−1^), alpha‐amino‐N is nitrogen (meq.100 g^−1^), WSC is white sugar content (%), WSY is white sugar yield (t. ha^−1^), and ESC is extraction coefficient of sugar (%).

### 2.3 Statistical analysis

Before any analysis, the homogeneity of the variances of experimental errors was checked with Bartlett's test (Bartlett, 1937). After the homogeneity of error variances was confirmed, a combined variance analysis was performed on each trait's data. Since the white sugar yield includes the values of other studied traits, it is considered a significant and final trait; Therefore, Additive Main Effect and Multiplicative Interactions (AMMI) stability analysis was performed in terms of this trait. Equation 6 was used to perform stability analysis by the AMMI method (Gauch, 1992):

(6) $$Y_{\mathrm{ge}}=\mu +\alpha_{g}+\beta_{e}+\sum_{n}\;\lambda_{n}\gamma_{\mathrm{gn}}\delta_{\mathrm{en}}+\rho_{\mathrm{ge}}$$

Where $Y_{\mathrm{ge}}\;$is the yield of genotype *g* in environment *e*; μ is the grand mean; $\alpha_{g}$ is the genotype deviation from the grand mean; $\beta_{e}$ is the environment deviation; $\lambda_{n}$ is the singular value for IPC_n_ and correspondingly $\lambda_{n}^{2}$ is its eigenvalue; $\gamma_{\mathrm{gn}}$ is the eigenvector value for genotype *g* and component *n*; $\delta_{\mathrm{en}}$ is the eigenvector value for environment *e* and component *n*, with both eigenvectors scaled as unit vectors; and $\rho_{\mathrm{ge}}$ is the residual. By performing AMMI analysis of variance using R software, the eigenvalues were obtained for each genotype and environment, and by drawing their biplots, the general and specific adaptability of the genotypes was determined. During this study, 13 statistics obtained from the AMMI model were used to identify the stable genotype in disease‐infected conditions through Equations 7 to 18:

(7) $$\text{AMGE}=\sum_{n=1}^{N}\;\lambda_{n}\gamma_{in}\delta_{jn}$$

(8) $$\mathrm{ASI}=\sqrt{\left[{\mathrm{PC}}_{1}^{2}\times \theta_{1}^{2}\right]+\left[{\mathrm{PC}}_{2}^{2}\times \theta_{2}^{2}\right]}$$

(9) $$\mathrm{ASV}=\sqrt{[\frac{\text{SSIPCA}1}{\text{SSIPCA}2}({\text{IPCA}}_{1\text{scrore})}]2+\left(\text{IPCA}2\text{scroe}\right)2}$$

(10) $$\text{STAB}=\sum_{n=1}^{N^{\prime }}\;\lambda_{n}\gamma_{in}^{2}$$

(11) $${\mathrm{AV}}_{\text{AMGE}}=\sum_{j=1}^{E}\;\sum_{n=1}^{N^{\prime }}\;\left|\lambda_{n}\gamma_{in}\delta_{jn}\right|$$

(12) $$\mathrm{Da}=\sqrt{\sum_{n=1}^{N}\;{\left(\lambda_{n}\gamma_{\text{in}}\right)}^{2}}$$

(13) $$D_{Z}=\sum_{n=1}^{N^{\prime }}\;\gamma_{in}^{2}$$

(14) $$\mathrm{EV}=\sum_{n=1}^{N}\;\frac{\gamma_{in}^{2}}{n}$$

(15) $$\mathrm{FA}=\sum_{n=1}^{N^{\prime }}\;\lambda_{n}^{2}\gamma_{in}^{2}$$

(16) $$\text{MASI}=\sqrt{\sum_{n=1}^{N\prime }\;{\mathrm{PC}}_{n}^{2}\times \theta_{1}^{2}}$$

(17) $$\text{MASV}=\sqrt{\sum_{n=1}^{N\prime }\left(\frac{{\text{SSIPC}}_{n}}{{\text{SSIPC}}_{n+1}}\right)\times {\left({\mathrm{PC}}_{n}\right)}^{2}+{\left({\mathrm{PC}}_{N^{\prime }}\right)}^{2}}\;$$

(18) $${\text{SIPC}}_{i}=\sum_{n=1}^{N}|\lambda_{n}^{0.5}\gamma |$$

(19) $$Z_{a}=\sum_{n=1}^{N^{\prime }}\;\left|\theta_{n}\gamma_{in}\right|$$

where AMGE is the sum across environments of GEI modeled by AMMI (Sneller et al., 1997), ASI is the AMMI stability index (Jambhulkar et al., 2014), ASV is the AMMI stability value (Purchase et al., 2000), ASTAB is AMMI‐based stability parameter (Rao & Prabhakaran, 2005), AV_AMGE_ is the sum across environments of the absolute value of GEI modeled by AMMI (Zali et al., 2012), Da is Annicchiarico's D parameter (Annicchiarico, 2002), Dz is Zhang's D parameter (Zhang et al., 1998), EV is the average of the squared eigenvector values (Zobel, 1994), FA is the stability measure based on fitted AMMI model (Raju, 2002), MASI is the modified AMMI stability index (Ajay et al., 2018), MASV is the modified AMMI stability value (Zali et al., 2012), SIPC is the sum of the absolute values of the IPC scores (Sneller et al., 1997), and Za is the absolute value of the relative contribution of IPCAs to the interaction (Zali et al., 2012).

MTSI index was computed to calculate the mean yield and simultaneous stability of root yield, sugar yield, sugar content, white sugar yield, white sugar content, sodium, potassium, alpha‐amino nitrogen, extraction coefficient of sugar, and molasses sugar based on Equation 20 (Olivoto et al., 2019):

(20) $${\text{MTSI}}_{i}={\left[\sum_{j=1}^{f}\;{\left(\gamma_{\mathrm{ij}}-\gamma_{j}\right)}^{2}\right]}^{0.5}$$

where ${\text{MTSI}}_{i}$ is the multi‐trait stability index of the genotype *i*, $\gamma_{\mathrm{ij}}$ is the score of the genotype *i* in the factor *j*, and $\gamma_{j}$ is the score of the ideal genotype in the factor *j*. Scores were calculated based on factor analysis for genotypes and traits.

## 3 RESULTS AND DISCUSSION

### 3.1 Assessment of genotypic response to disease

The results of examining the reaction of genotypes against rhizomania disease based on the Luterbacher et al. (2005) method are given in Table 3. Examining the frequency of sensitivity/resistance of genotypes against rhizomania in each of the research stations for 2 years showed that the genotypes are different in terms of reaction to this disease; the majority of them had a response in the semi‐resistant to semi‐sensitive range. According to the evaluation of genotypes against the disease in Mashhad in 2018, five of the genotypes under study, including RM1, RM7, RM10, RM14, and RM19, along with two controls, Baloo and BTS335, had healthy roots and no hairy root or color change; in other words, they had a complete resistance reaction. Therefore, these genotypes can carry genes that are involved in the observed resistance. Regarding the reaction of genotypes to rhizomania in Mashhad in 2019, only one genotype (RM17) showed a complete resistance reaction, and the other genotypes along with the controls showed a range of semi‐resistant to semi‐sensitive reactions. As is evident in the results, the genotypes that were completely resistant in 2018 showed different degrees of semi‐resistant to semi‐sensitive reaction in 2019. The reason can be attributed to the environmental conditions and the development of new isolates of the disease, which caused the absence of complete resistance of genotypes. Based on the results obtained from the infection severity of the genotypes to the disease in Shiraz, none of the studied genotypes had a complete resistance reaction to the disease during the 2 years of the experiment, and they had a reaction in the semi‐resistant to semi‐sensitive range. What is certain is that during both years, the infection severity of genotypes in Shiraz was higher than that in Mashhad. Therefore, it can be acknowledged that the environmental conditions in Shiraz for the development and establishment of more virulent isolates of rhizomania were more favorable than the environmental conditions in Mashhad; Therefore, genotypes should be recommended for cultivation in Shiraz that have more effective resistance genes against the disease to prevent its development and causing heavy damages due to the reduction of sugar content and root yield.

**TABLE 3:** Reaction of sugar beet genotypes against rhizomania in each of the agricultural research stations

Genotype	Mashhad		Shiraz		Genotype	Mashhad		Shiraz	
	2018	2019	2018	2019		2018	2019	2018	2019
RM1	1	2	4	4	RM14	1	2	5	3
RM2	3	2	4	3	RM15	2	2	4	3
RM3	2	2	4	3	RM16	2	2	5	2
RM4	2	3	3	3	RM17	2	1	4	3
RM5	3	3	4	3	RM18	2	2	4	3
RM6	3	2	6	4	RM19	1	3	4	3
RM7	1	3	6	3	RM20	2	2	4	2
RM8	2	4	4	3	SBSI038	3	3	6	6
RM9	2	4	5	3	Lexia	2	2	4	3
RM10	1	2	4	3	Baloo	1	4	5	5
RM11	2	2	4	3	Poseidon	3	5	4	4
RM12	2	2	5	3	BTS335	1	2	5	3
RM13	2	4	5	2	‐	‐	‐	‐	‐

### 3.2 Combined analysis of variance

Bartlett's test (Bartlett, 1937) confirmed the uniformity of error variances in different trials. Therefore, to determine the genotype × environment interaction on the data obtained from the root yield, sugar yield, sugar content, white sugar content, white sugar yield, Na^+^, K^+^, alpha‐amino N, molasses sugar, and extraction coefficient of sugar, a combined analysis of variance was performed (Table 4). The main effects of year, location, and genotype for all the mentioned traits were significant at 1% probability level. Two‐way interactions of year × location (except for K^+^), year × genotype, and location × genotype were significant for all studied traits at 1% probability level. The three‐way interaction of genotype × year × location was significant at 1% and 5% probability levels for all studied traits. The significance of the interactions is due to the large variations in genotypes across the years and locations under investigation, as well as variation in the relative rank of genotypes.

**TABLE 4:** Combined analysis of variance for the studied traits of sugar beet genotypes

Source of variation	Df	Mean of squares				
		Root yield	Sugar yield	Sugar content	White sugar content	White sugar yield
Year	1	1947.2**	240.8**	93.9**	41.2**	166.5**
Location	3	42527.8**	2404.5**	680.6**	1281.6**	2943.8**
Year × Location	3	35118.8**	953.8**	262.3**	344.8**	686.8**
Error 1	24	216.9	10.3	2.8	4.9	9.3
Genotype	24	2112.9**	79.8**	14.3**	24.9**	64.2**
Genotype × Year	24	326.9**	10.9**	1.8**	2.4**	8.6**
Genotype × location	72	365.2**	15.6**	2.7**	4.3**	13.1**
Genotype × Year × location	72	263.9**	9.0**	1.4**	2.0**	7.4**
Error 2	576	74.1	2.8	0.9	1.2	2.3

Source of variation	Df	Mean of squares				
		Na^+^	K^+^	Alpha‐amino nitrogen	Molasses sugar	Extraction coefficient of sugar
Year	1	5.8**	22.4**	75.8**	10.6**	2.8**
Location	3	593.1**	231.1**	19.3**	183.9**	9181.2**
Year × Location	3	48.2**	2.9 ns	19.6**	6.8**	1422.6**
Error 1	24	0.8	3.4	0.4	0.6	30
Genotype	24	8.1**	3.0**	1.3**	2.0**	142.5**
Genotype × Year	24	07**	0.5**	0.2**	0.2**	13.3**
Genotype × location	72	1.6**	0.6**	0.2**	0.3**	33.1**
Genotype × Year × location	72	0.6**	0.3*	0.1**	0.1**	12.7**
Error 2	576	0.3	0.2	0.1	0.1	6.2

*Note*: *, **Significant at 5% and 1% probability levels, respectively.

Abbreviation: ns, nonsignificant.

### 3.3 AMMI analysis

According to the AMMI model results (Table 5), the genotype × year × location interaction for white sugar yield was significant at 1% probability level. The percentage of variance explained by genotype × year × location interaction was equal to 10.65%. In a study conducted on maize (*Zea mays* L.) using the AMMI method, the variance explained by the genotype × environment interaction was estimated as 7.84% (Basafa & Taherian, 2016). The multiplicative effect of AMMI model was decomposed into interaction principal components. Based on these results (Table 5), the first five components had a significant interaction at 1% probability level, and the sixth component had a significant interaction at 5% probability level. The first component explained 45.50% of the interaction variations. The second to sixth principal components were able to explain 19.20%, 14.60%, 9.30%, 5.40%, and 4.80% of the variations related to the genotype × year × location interaction, respectively. These components together with the first component explained 98.80% of the total variations of genotype × year × location interaction. The residual sum of squares (Noise) from the AMMI model with the lowest mean of square was found to be nonsignificant, which indicates the considerable accuracy of this model (Anandan & Eswaran, 2009). In a study conducted using the AMMI model, Mostafavi and Saremirad (2021) stated that the first principal component of the interaction was significant and explained about 63% of the data variation. Karimizadeh et al. (2008) showed that the five main components of the interaction explained 90.30% of the variations of genotype × environment interaction. Omrani et al. (2019) showed that the first four components together explained 83% of the genotype × environment interaction variations.

**TABLE 5:** Analysis of variance of genotype × environment interaction for white sugar yield of sugar beet genotypes based on AMMI model

Source of variation	Df	Sum of squares	Mean of squares	Relative variance (%)	Cumulative variance (%)
IPCA1	30	768.50	25.61**	45.50	45.50
IPCA2	28	325.02	11.60**	19.20	64.70
IPCA3	26	246.56	9.48**	14.60	79.30
IPCA4	24	157.85	6.57**	9.30	88.60
IPCA5	22	91.98	4.18*	5.40	94.10
IPCA6	20	80.30	4.01*	4.80	98.80
Noise	18	19.93	1.10^ns^	1.20	100.00

*Note*: ns, *, **: nonsignificant and significant at 5% and 1% probability levels, respectively.

**FIGURE 1:**
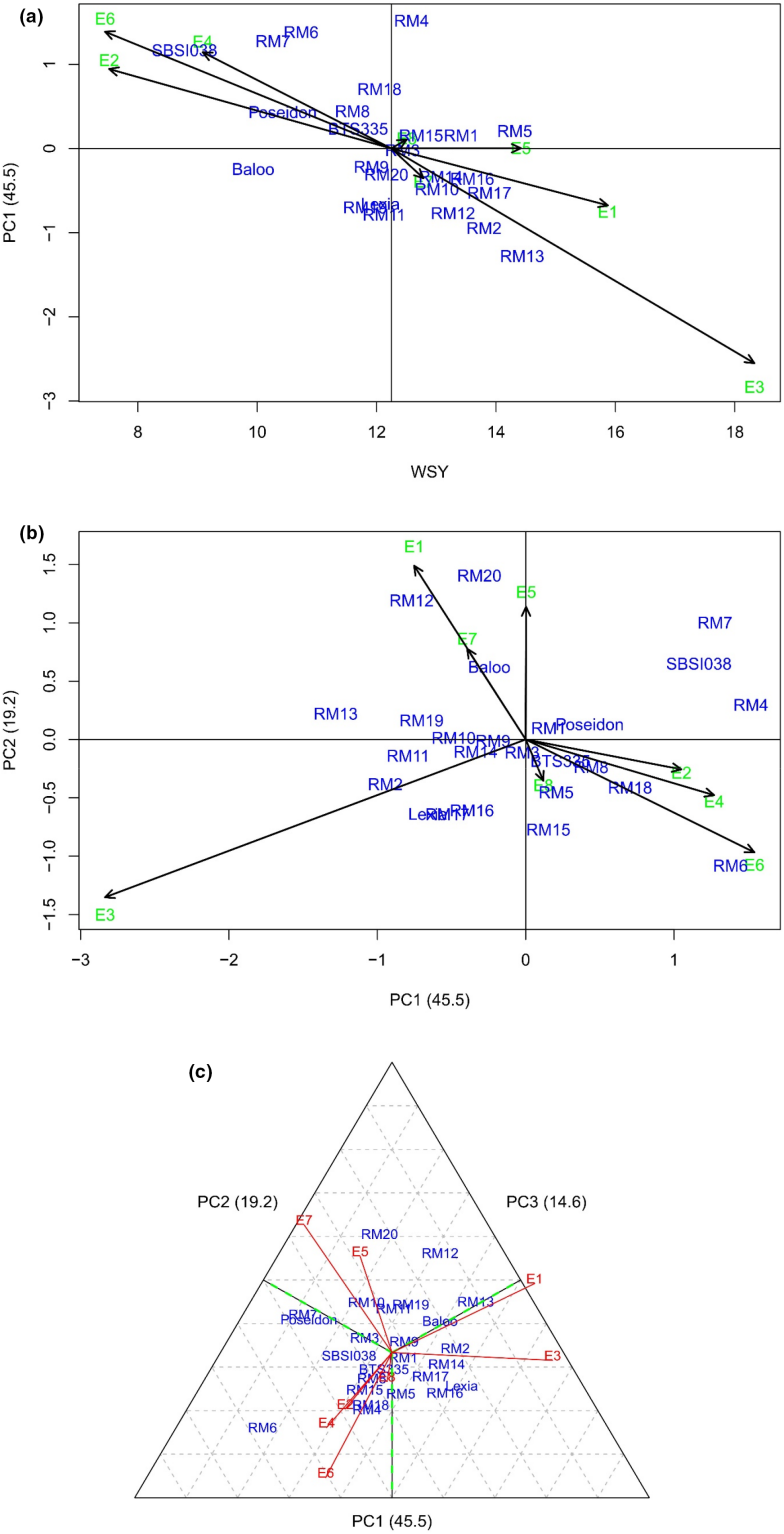
(a) Scatter plot for genotypes and environments derived from yield means and a first principal component; (b) Scatter plot for genotypes and environments derived from the first two interaction principal components; (c) Scatter plot for genotypes and environments derived from the first three interaction principal components.

To consider yield stability and specific adaptation of genotypes to the studied areas, the biplot of white sugar yield with the first principal component (Figure 1a) and a biplot of the two first principal components (Figure 1b) was used. Also, for maximum certainty, the biplot of the first three main components explaining about 80% of the variations was used. According to the biplot of average white sugar yield versus the first principal component of the interaction, the genotype that has a higher amount in terms of white sugar yield (horizontal axis) and a lower value in terms of the first component of the genotype × environment interaction (vertical axis) will be more favorable. Based on this, among the genotypes, RM5, and among the environments, Mashhad in 2019 were recognized as the most stable genotype and environment, respectively, due to its white sugar yield being higher than the total average and the low value of the first interaction component. If the genotype and environment have the same sign in terms of the first component of the interaction, they will have a positive interaction, and if they do not have the same sign in terms of the mentioned component, they will have a negative interaction. Mashhad and Miandoab environments with white sugar yields above the total average had positive interaction with RM13, RM2, RM12, RM11, RM19, Lexia, RM17, RM16, RM14, RM20, RM9, Baloo, RM10, and RM3 and negative interaction with RM4, RM6, RM7, SBSI038, RM18, RM8, Poseidon, BTS335, RM1, RM5, and RM15. The environments of Shiraz and Hamadan had a situation opposite to that of Mashhad and Miandoab so that they showed a similar interaction. These environments interacted positively with RM4, RM6, RM7, SBSI038, RM18, RM8, Poseidon, BTS335, RM1, RM5, and RM15 and negatively with RM13, RM2, RM12, RM11, RM19, Lexia, RM17, RM16, RM14, RM20, RM9, Baloo, RM10, and RM3.

Figure 1b shows the values of the first and second principal components of the genotype × environment interaction for genotypes and environments. A total of 64.70% of the variations related to the multiplicative effect was explained by this biplot. According to this biplot, the genotypes that are located in the vicinity of a place have specific adaptation to that environment, whereas the genotypes that are close to the origin of the coordinates have general adaptation. Accordingly, there is considerable specific adaptation between RM13 with Mashhad (2018) and Miandoab (2018), RM5 with Mashhad (2019), RM11 and RM10 with Miandoab (2019), RM15 with Hamedan (2019), and SBSI038 with Hamedan (2018), Shiraz (2018) and Shiraz (2019). RM3 followed by RM15, BTS335, and RM9 had general adaptation due to being close to the origin of coordinates. To ensure the maximum reliability of the results, the biplot of the first three principal components (Figure 1c) was also used to identify genotypes with high general adaptability, justifying about 80% of the variations. Based on the results of this biplot, RM9 with an average white sugar yield of 11.91 t. ha^−1^ was recognized as a genotype with wide general adaptability because it had values of three components close to zero. None of the environments under study had values of the first, second, and third components of the genotype × environment interaction close to zero (origin of coordinates), which indicates that the environments have the potential to create interaction.

The results of the average white sugar yield of genotypes and different stability statistics of AMMI analysis can be seen in Table 6. The average white sugar yield of genotypes in all environments was estimated at 12.24 t. ha^−1^. RM13 and RM5 had the highest white sugar yields of 14.44 and 14.31 t. ha^−1^, respectively. The lowest white sugar yield belonged to SBSI038 and Baloo with values of 8.78 and 9.93 t. ha^−1^, respectively. The white sugar yield of RM20, RM11, and RM3 was within the average white sugar yield of all genotypes in all environments with values of 12.16, 12.14, and 12.43 t. ha^−1^, respectively. According to the AMGE stability index, the highest stability with a lower amount of this statistic was observed in RM18. Based on ASI and MASI statistics, RM3 was the most stable with the lowest values for these two statistics. Meanwhile, RM4 was known as the most unstable genotype. The results obtained using ASTAB, AVAMGE, DA, DZ, EV, FA MASV, SIPC, and Za statistics indicated that RM9 and BTS335 with the lowest values for these statistics were the most stable genotypes. Based on the mentioned statistics, RM6, RM4, Baloo, and RM20 with the highest values were identified as the most unstable genotypes. The results of the present study were somewhat similar to the findings of Cheloei et al. (2020); Karimizadeh et al. (2016); and Sharifi et al. (2017). They acknowledged that the most accurate model in the AMMI decomposition could be predicted using the first two principal components. Despite the different stability analysis methods, the AMMI model provides useful information to achieve accurate results (Sharifi et al., 2017). Based on the results of the present study, the majority of stable genotypes based on different AMMI stability statistics had an average white sugar yield around the total average. Meanwhile, Ajay et al. (2020) reported that according to these 12 AMMI stability statistics (especially SIPC, MASI, and MASV statistics), high‐yielding genotypes can be identified.

**TABLE 6:** White sugar yield mean and different AMMI stability parameters in the studied sugar beet genotypes

Genotype	White sugar yield mean (t. ha^−1^)	AMGE	ASI	ASV	ASTAB	AVAMGE	DA	DZ	EV	FA	MASI	MASV	SIPC	Za
RM1	13.42	3.3 E‐15	0.07	0.38	0.55	4.64	1.93	0.29	0.01	3.72	0.10	1.20	1.56	0.07
RM2	13.80	2.11 E‐15	0.44	2.27	2.22	9.86	4.46	0.56	0.05	19.88	0.44	2.69	3.38	0.20
RM3	12.43	6.8 E‐16	0.02	0.13	0.72	4.54	2.15	0.34	0.02	4.64	0.09	1.26	1.76	0.07
RM4	12.58	2.82 E‐15	0.69	3.60	3.53	14.76	6.46	0.57	0.05	41.67	0.71	3.98	3.01	0.26
RM5	14.31	1.4 E‐15	0.13	0.66	0.54	4.55	2.06	0.27	0.01	4.23	0.14	1.06	1.54	0.09
RM6	10.73	‐2.83 E‐15	0.66	3.44	4.17	14.82	6.73	0.64	0.07	45.28	0.68	3.92	3.94	0.31
RM7	10.26	−5.5 E‐15	0.61	3.18	2.83	11.25	5.74	0.51	0.04	32.98	0.61	3.36	3.02	0.25
RM8	11.59	−4.3 E‐15	0.21	1.07	0.78	4.93	2.50	0.34	0.02	6.25	0.21	1.56	1.92	0.11
RM9	11.91	4.11 E‐15	0.10	0.52	0.32	2.95	1.43	0.24	0.01	2.04	0.11	0.84	1.05	0.05
RM10	13.01	3.08 E‐15	0.22	1.15	1.55	7.50	3.59	0.45	0.03	12.87	0.27	2.16	2.30	0.14
RM11	12.14	−5.55 E−16	0.36	1.87	1.83	10.53	4.04	0.50	0.04	16.29	0.38	2.41	2.95	0.18
RM12	13.28	‐1.29 E‐14	0.42	2.18	3.21	12.32	5.23	0.66	0.07	27.31	0.43	2.81	3.82	0.23
RM13	14.44	−5.61 E‐15	0.58	3.04	2.32	12.18	5.16	0.49	0.04	26.59	0.59	3.22	2.89	0.22
RM14	13.07	3 E‐15	0.15	0.80	2.22	8.41	3.69	0.63	0.07	13.60	0.20	2.13	2.94	0.14
RM15	12.74	2.55 E‐15	0.16	0.85	0.88	6.20	2.71	0.33	0.02	7.33	0.17	1.30	1.88	0.10
RM16	13.60	−6.44 E‐15	0.20	1.05	1.88	8.55	3.79	0.51	0.04	14.35	0.24	2.28	2.77	0.16
RM17	13.89	3.89 E‐16	0.27	1.40	0.75	5.83	2.82	0.27	0.01	7.96	0.27	1.55	1.64	0.12
RM18	12.05	−9.99 E‐16	0.33	1.71	0.82	7.68	3.08	0.28	0.01	9.48	0.33	1.85	1.82	0.14
RM19	11.81	−6.42 E‐15	0.32	1.67	1.19	8.32	3.30	0.40	0.03	10.90	0.33	2.02	2.38	0.15
RM20	12.16	−3.89 E‐15	0.30	1.59	3.40	12.61	5.14	0.69	0.08	26.38	0.32	2.45	3.58	0.19
SBSI038	8.78	−4.44 E‐15	0.55	2.84	2.40	11.22	5.14	0.50	0.04	26.38	0.55	3.10	3.31	0.24
Lexia	12.06	1.1 E‐14	0.32	1.69	2.10	10.02	4.22	0.53	0.05	17.84	0.35	2.51	3.20	0.20
Baloo	9.93	1.16 E‐14	0.16	0.85	3.18	9.81	4.38	0.76	0.10	19.16	0.21	2.38	3.72	0.17
Poseidon	10.43	2.1 E‐15	0.20	1.03	2.56	9.98	4.26	0.63	0.07	18.13	0.26	2.47	3.45	0.17
BTS335	11.69	2.11 E‐15	0.11	0.58	0.34	3.81	1.51	0.24	0.01	2.29	0.12	0.84	1.08	0.06

Abbreviations: AMGE, Sum Across Environments of GEI Modeled by AMMI; ASI, AMMI Stability Index; ASV, AMMI Stability Value; ASTAB, AMMI‐Based Stability Parameter; AVAMGE, Sum Across Environments of Absolute Value of GEI Modeled by AMMI; DA, Annicchiarico's D Parameter; DZ, Zhang's D Parameter; EV, Averages of the Squared Eigenvector Values; FA, Stability Measure Based on Fitted AMMI Model; MASI, Modified AMMI Stability Index; MASV, Modified AMMI Stability Value; SIPC, Sums of the Absolute Value of the IPC Scores; Za, Absolute Value of the Relative Contribution of IPCAs to the Interaction.

### 3.4 MTSI analysis

Factor analysis was done based on principal component analysis, and interpretation of results was performed after Varimax rotation. The results of factor analysis are presented in Table 7. Factors with eigenvalues greater than 1 were selected, and the variance of each factor was expressed as a percentage, which indicates its importance in interpreting the overall variations in the data. In this analysis, three independent factors explained 90.83% of data variation. The first factor explained 42.79% of data variance and had an eigenvalue of 3.85. This factor had high and negative coefficients for white sugar content, Na^+^, K^+^, sugar content, extraction coefficient of sugar, and molasses sugar. The second factor with an eigenvalue of 2.56 and justification of 28.53% of total variance, included high and positive factor coefficients for root yield, white sugar yield, and sugar yield. The third factor explained 12.57% of data variation, and with an eigenvalue of 1.13 displayed a high and negative factor coefficient for alpha‐amino N. The MTSI stability index of the studied genotypes was calculated based on factor scores of the three mentioned factors.

**TABLE 7:** Eigenvalues, relative and cumulative variance as well as factor coefficients after varimax rotation in factor analysis based on principal component analysis

Traits	Factors		
	First	Second	Third
Root yield	0.149	0.970	−0.020
Sugar yield	−0.147	0.983	0.026
Sugar content	−0.866	0.049	0.262
White sugar content	−0.944	−0.015	0.111
White sugar yield	−0.264	0.953	0.004
Na^+^	−0.668	0.145	−0.359
K^+^	−0.643	0.154	−0.359
alpha‐amino nitrogen	−0.013	−0.064	−0.858
Molasses sugar	−0.919	−0.174	0.001
Extraction coefficient of sugar	−0.923	0.076	−0.222
Eigenvalue	3.85	2.56	1.13
Relative variance (%)	42.79	28.53	12.57
Cumulative variance (%)	42.79	71.32	83.90

**FIGURE 2:**
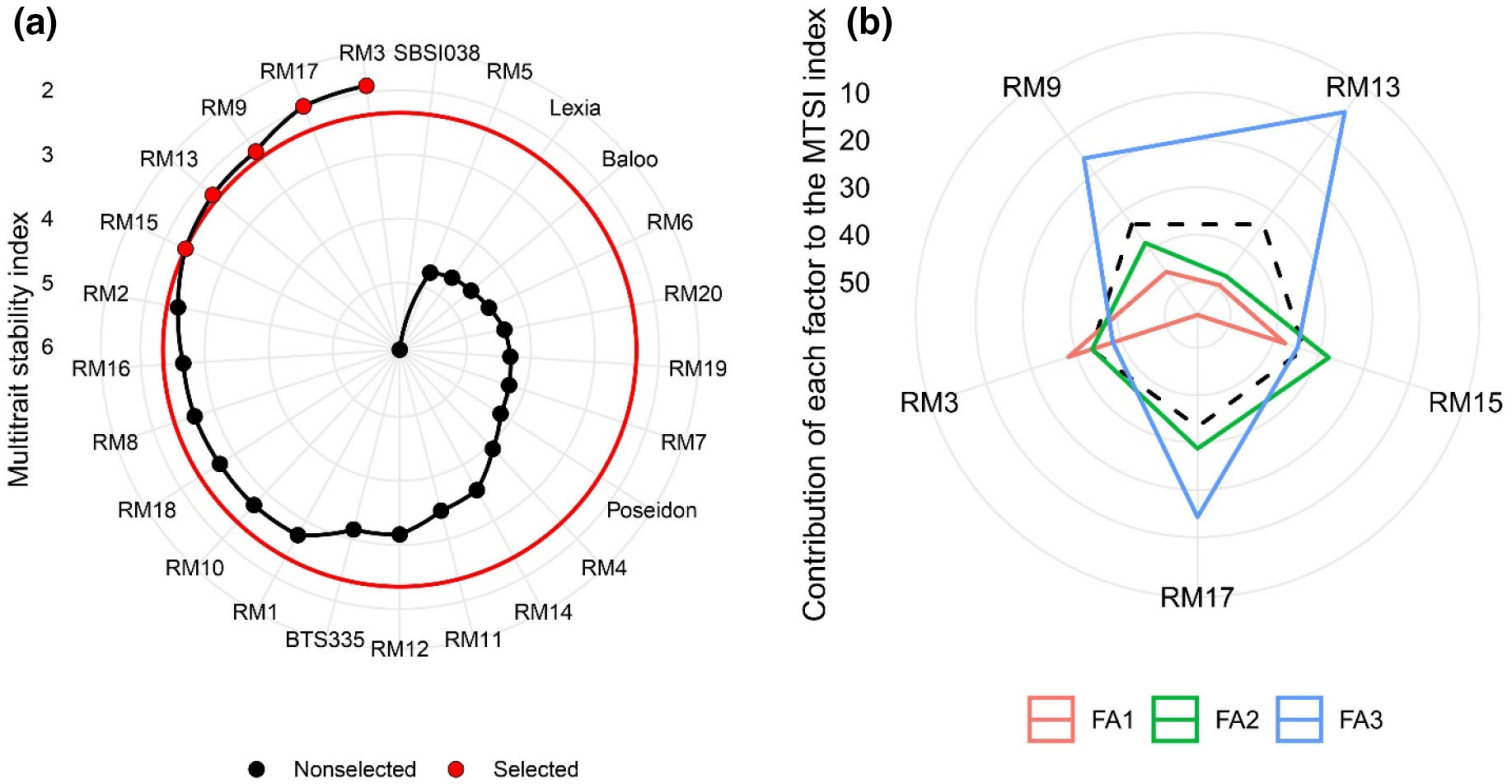
(a) Ranking of genotypes in ascending order based on MTSI index and (b) Strengths and weaknesses of selected genotypes as the ratio of each factor in the calculated MTSI index.

According to the MTSI index, if the genotype value is less than this index, it is less distant from the ideal genotype. On the other hand, if the MTSI value is higher for the genotype, it means that it is more distant from the ideal genotype and should not be selected (Olivoto et al., 2019). In Figure 2a, the experimental genotypes are ranked from the highest to the lowest value of the MTSI index so that the genotype with the highest value of MTSI is in the center and the genotype with the lowest value of MTSI is located in the outermost circuit. Based on this, by applying a selection pressure of 20%, RM3 ranked first and RM17, RM9, RM13, and RM15 ranked next as the most ideal stable genotypes in terms of all traits. Comparison of the value of traits in the selected genotypes based on MTSI with other genotypes showed that the mean value of root yield, sugar yield, white sugar yield, white sugar content, and extraction efficiency of sugar has been increased in selected genotypes. This increase in the value of traits was aimed at the intended goals. The goal that is followed in Na^+^, K^+^, alpha‐amino N, and molasses sugar is to reduce their value, and the selected genotypes showed have a lower value in terms of these traits. In general, the selected genotypes caused a favorable selection differential in all traits except sugar content and white sugar content (Table 8). In the selected genotypes, all traits had high heritability. SBSI038 genotype had the highest value of the MTSI stability index.

**TABLE 8:** Prediction of selection differential and heritability for effective traits based on MSTI index

Factors	Traits	Goal	Xo	Xs	SD	SD percent	*h* ^2^
1	Sugar content	Increase	18.68	18.61	−0.07	−0.42	0.85
1	Na^+^	Decrease	2.20	2.09	−0.11	−5.04	0.86
1	K^+^	Decrease	4.97	4.83	−0.14	−2.84	0.82
1	White sugar content	Increase	15.78	15.80	0.01	0.09	0.87
1	Extraction coefficient of sugar	Increase	83.81	84.41	0.60	0.72	0.84
1	Molasses sugar	Decrease	2.30	2.20	−0.09	−4.24	0.76
2	Root yield	Increase	76.69	81.84	5.15	6.71	0.85
2	Sugar yield	Increase	14.39	15.29	0.90	6.28	0.84
2	White sugar yield	Increase	12.24	13.08	0.83	6.84	0.84
3	alpha‐amino nitrogen	Decrease	1.58	1.51	−0.07	−4.84	0.85

Abbreviations: SD, selection differential; SD perc, selection differential in percentage; SG, selection gain; SG perc, selection gain in percentage; Xo, original value; Xs, selected value.

Figure 2b presents the strengths and weaknesses of the selected genotypes based on the contribution of each factor in the MTSI index. The smallest the proportion explained by a factor (closer to the external edge), the closer the traits within that factor are to the ideotype (Olivoto et al., 2021). The dashed line shows the theoretical value if all the factors had contributed equally (Olivoto et al., 2021). RM3 and RM15, which had the lowest value in the first factor for sugar content, Na^+^, K^+^, white sugar content, extraction coefficient of sugar, and molasses sugar, and the highest factor coefficients in this factor, are close to the ideal genotype. The ideal genotype is defined according to the traits contained in each factor and the goals that are intended to improve those traits. RM15, RM17, RM3, and RM9 had the lowest contribution to the second factor. As a result, these genotypes were very close to the ideal genotype in terms of root yield, white sugar yield, and sugar yield. In other words, these genotypes had high values of root yield, sugar yield, and white sugar yield. RM13, RM17, and RM9 had high strength to the third factor. In other words, they had low alpha‐amino N values and stability. These results were consistent with the findings of Sharifi et al. (2021) who used the MTSI stability index for the evaluation of yield and other agronomic traits in a set of rice genotypes and showed that this index is well able to identify superior genotypes in terms of yield stability and other agronomic traits.

## 4 CONCLUSION

Rhizomania disease is one of the main factors in reducing quantitative and qualitative yield of sugar beet. Breeding genotypes with disease resistance genes is one of the most important solutions to deal with this destructive disease. Identifying sources of resistance and including them in breeding programs will improve resistant genotypes. For this purpose, in the present study, the resistance level of sugar beet genotypes against rhizomania disease was investigated in terms of the infection severity and quantitative and qualitative traits. The analysis of variance for the studied traits in different areas infected with the disease showed significant genetic diversity among the genotypes. The results of the genotypes evaluation in terms of reaction to rhizomania disease showed that the number of genotypes with resistance reaction is much more than the number of genotypes with sensitivity reaction. Examining the genotypes yield stability in disease‐infected environments by different statistics introduced somewhat different genotypes as stable ones in terms of white sugar yield, but overall, the majority of stability analysis methods agreed on the stability of RM18, RM3, RM9, and BTS335; However, the MTSI stability statistic considering all the traits presented somewhat different results and introduced RM3, RM17, RM9, RM13, and RM15 as stable genotypes in terms of all the investigated traits under disease‐infected conditions.

## FUNDING INFORMATION

This work was supported by the Sugar Beet Seed Institute (SBSI), Karaj, Alborz, Iran. The funding body was involved in the material creation.

## CONFLICT OF INTEREST

The authors report no conflicts of interest in this work. The authors alone are responsible for the content and writing of this article.

## Data Availability

The data that support the findings of this study are available from the corresponding author upon reasonable request.
